# Effect of *tcdR* Mutation on Sporulation in the Epidemic *Clostridium difficile* Strain R20291

**DOI:** 10.1128/mSphere.00383-16

**Published:** 2017-02-15

**Authors:** Brintha P. Girinathan, Marc Monot, Daniel Boyle, Kathleen N. McAllister, Joseph A. Sorg, Bruno Dupuy, Revathi Govind

**Affiliations:** a Division of Biology, Kansas State University, Manhattan, Kansas, USA; b Department of Microbiology, Laboratoire Pathogenèse des Bactéries Anaérobies, Institut Pasteur, Paris, France; c Department of Biology, Texas A&M University, College Station, Texas, USA; University of Iowa

**Keywords:** *Clostridium difficile*, sporulation, toxin gene regulation

## Abstract

*Clostridium difficile* is an important nosocomial pathogen and the leading cause of hospital-acquired diarrhea. Antibiotic use is the primary risk factor for the development of *C. difficile*-associated disease because it disrupts normally protective gut flora and enables *C. difficile* to colonize the colon. *C. difficile* damages host tissue by secreting toxins and disseminates by forming spores. The toxin-encoding genes, *tcdA* and *tcdB*, are part of a pathogenicity locus, which also includes the *tcdR* gene that codes for TcdR, an alternate sigma factor that initiates transcription of *tcdA* and *tcdB* genes. We created a *tcdR* mutant in epidemic-type *C. difficile* strain R20291 in an attempt to identify the global role of *tcdR*. A site-directed mutation in *tcdR* affected both toxin production and sporulation in *C. difficile* R20291. Spores of the *tcdR* mutant were more heat sensitive than the wild type (WT). Nearly 3-fold more taurocholate was needed to germinate spores from the *tcdR* mutant than to germinate the spores prepared from the WT strain. Transmission electron microscopic analysis of the spores also revealed a weakly assembled exosporium on the *tcdR* mutant spores. Accordingly, comparative transcriptome analysis showed many differentially expressed sporulation genes in the *tcdR* mutant compared to the WT strain. These data suggest that regulatory networks of toxin production and sporulation in *C. difficile* strain R20291 **a**re linked with each other.

**IMPORTANCE** *C. difficile* infects thousands of hospitalized patients every year, causing significant morbidity and mortality. *C. difficile* spores play a pivotal role in the transmission of the pathogen in the hospital environment. During infection, the spores germinate, and the vegetative bacterial cells produce toxins that damage host tissue. Thus, sporulation and toxin production are two important traits of *C. difficile*. In this study, we showed that a mutation in *tcdR*, the toxin gene regulator, affects both toxin production and sporulation in epidemic-type *C. difficile* strain R20291.

## Abstract (precis)

*C. difficile* infects thousands of hospitalized patients every year, causing significant morbidity and mortality. *C. difficile* spores play a pivotal role in the transmission of the pathogen in the hospital environment. During infection, the spores germinate, and the vegetative bacterial cells produce toxins that damage host tissue. Thus, sporulation and toxin production are two important traits of *C. difficile*. In this study, we showed that a mutation in *tcdR*, the toxin gene regulator, affects both toxin production and sporulation in epidemic-type *C. difficile* strain R20291.

## INTRODUCTION

*Clostridium difficile* is a Gram-positive, spore-forming, anaerobic bacillus and is the leading cause of hospital-acquired diarrheal diseases (1, 2). Nearly 50% of all patients carry *C. difficile* asymptomatically after hospitalization (2, 3). Nearly 10% of all *C. difficile*-infected patients develop pseudomembranous colitis, and 3% develop severe, life-threatening complications such as fulminant colitis and toxic megacolon (4). *C. difficile* infection (CDI) is commonly acquired from *C. difficile* spores present in the hospital environment, and individuals become infected when the normal colonic microbiota is suppressed by antibiotic therapy (5). In the gut, *C. difficile* spores germinate to the toxin-producing vegetative form in response to certain bile acids, e.g., taurocholic acid (TA), and amino acids. *C. difficile* toxins A (TcdA) and B (TcdB) are then secreted from the vegetative cell and cause tissue damage, necrosis, and inflammation and are the main reasons for this disease outcome (6).

In *C. difficile*, the toxin genes, *tcdA* and *tcdB*, are located within a 19-kb pathogenicity locus (PaLoc) and the *tcdR* gene, located upstream of *tcdB*, is required for expression of the toxin genes. TcdR is an alternate sigma factor that directs transcription by recruiting RNA polymerase to the toxin gene promoters and its own promoter (7, 8). Previous studies have shown that other proteins can regulate toxin gene expression in response to different environmental stimuli by controlling the transcription of *tcdR*. The sigma factor SigD positively regulates toxin production by controlling the transcription of *tcdR* (9). CodY, a global transcriptional regulator, represses the toxin gene expression by binding with high affinity to the *tcdR* promoter region (10, 11). Finally, in response to sugar availability, CcpA, a major regulator of carbon catabolite repression, binds to the promoter region or the 5′ ends of several PaLoc genes, with the strongest affinity to the promoter region of *tcdR* (12, 13).

TcdR was the first member of the group V family of alternative sigma factors to be described (14). We recently determined that TcsR, a toxin gene regulator in *Clostridium sordellii*, is also a member of this family of sigma factors (15). Most of these alternative sigma factors are autoregulated (7, 16) and are induced by environmental stresses, such as nutritional limitation, DNA damage, or nonoptimal temperatures (8, 14, 17), suggesting that these sigma factors function under these suboptimal growth conditions.

In this study, we created and characterized a mutation in *tcdR* in the epidemic-type *C. difficile* R20291 strain to determine whether TcdR influenced cellular processes other than toxin production. We found that the *tcdR* mutant sporulated less efficiently than the wild-type (WT) strain. Moreover, spores prepared from the *tcdR* mutant were more heat sensitive and had lower germination efficiency than the wild-type parental strain. Electron microscopic (EM) analysis of the *tcdR* mutant spores also revealed a weakly assembled exosporium. In agreement with these findings, comparative transcriptome sequencing (RNA-seq) analyses of the WT and the *tcdR* mutant strains revealed several sporulation genes to be affected by the *tcdR* mutation. These results suggested that a mutation in *tcdR* not only affects toxin production but also influences the sporulation pathway in the *C. difficile* R20291 strain. Interestingly, however, mutating *tcdR* in the *C. difficile* 630Δ*erm* strain did not result in this phenotype, suggesting that the TcdR regulon may be strain specific.

## RESULTS

### Mutation in *tcdR* affects both toxin production and sporulation in *C. difficile* strain R20291.

**FIG 1:**
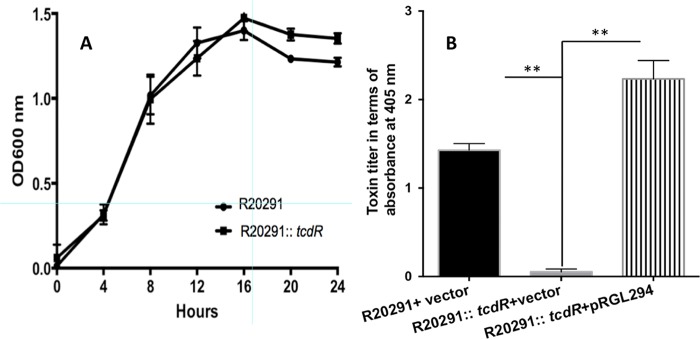
Effect of *tcdR* inactivation on bacterial growth kinetics and toxin production. (A) Growth curve of R20291 and R20291::*tcdR* in TY medium. (B) TcdA and TcdB levels in cytosolic fractions after 10 h of growth. *C. difficile* strains were grown in TY medium, and toxins were quantified using ELISA. The data represent the averages of the results of three independent assays. Error bars in both panel A and B correspond to the standard errors of the means. The asterisks (**) in panel B indicate statistical difference at a *P* value of <0.005.

**FIG 2:**
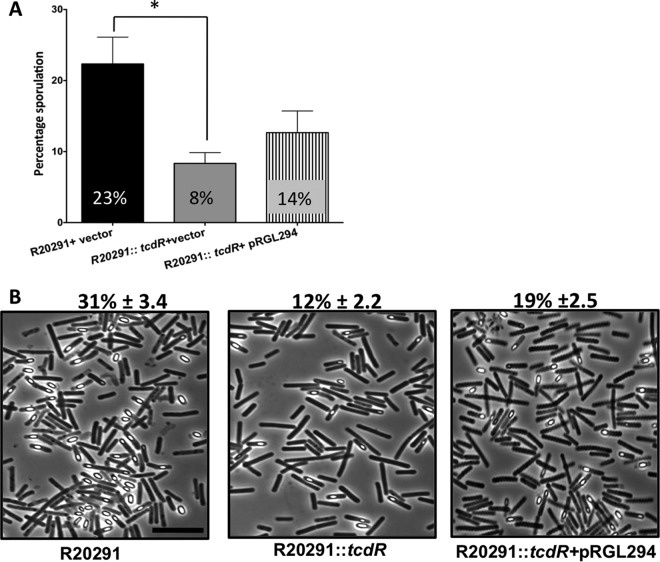
Mutation in *tcdR* affects the sporulation efficiency in the R20291 strain. (A) Sporulation frequency (CFU per milliliter of ethanol-resistant spores) of R20291 plus pRPF185 (R20291+pRPF185), R20291::*tcdR*+pRPF185, and R20291::*tcdR*+pRGL294 (pRPF185 derivative plasmid carrying *tcdR*) strains grown for 24 h in 70:30 sporulation medium. The error bars correspond to standard errors of the means of results from 3 biological replicates. *, *P* < 0.05 (by two-tailed Student’s *t* test). At least three independent experiments were performed. (B) Phase-contrast microscopy of paraformaldehyde-fixed R20291, R20291::*tcdR*+pRPF185, and R20291::*tcdR*+pRGL294 strains grown for 24 h in 70:30 sporulation plate. Percent sporulation (± standard deviation) was calculated (using the number of spores divided by the total number of spores and vegetative cells) from results from at least three independent experiments. Bar, 10 µm.

To analyze the global role of *tcdR* in *C. difficile* strain R20291, we used a Clostron system (18) to inactivate the *tcdR* gene. Insertion of the group II intron into the target gene (see Fig. S1A in the supplemental material) was verified by PCR using intron-specific primers and *tcdR* gene-specific primers (Fig. S1B and Table S1 in the supplemental material). Southern blotting confirmed the single chromosomal insertion of the intron in the *tcdR* gene (Fig. S1C). Growth kinetics analyses were performed and indicated that the inactivation of the *tcdR* gene did not affect the normal growth of the bacterium (Fig. 1A). A toxin enzyme-linked immunosorbent assay (ELISA) was performed with the cytosolic protein extracts of the *tcdR* mutant and the WT strain. We observed a dramatic reduction in toxin production (Fig. 1B) in the mutant compared to the WT, supporting the concept of the previously known function of TcdR as a positive regulator of the toxin genes (7, 8, 16). Further, we measured the sporulation efficiency of the *tcdR* mutant at the 24-h time point. A nearly 3-fold reduction in the level of ethanol-resistant spores was observed in the *tcdR* mutant compared to the WT strain (Fig. 2A). A similarly reduced sporulation rate (~2.6-fold) was observed when the number of sporulation cells in the population was counted microscopically (Fig. 2B). We then complemented the *tcdR* mutant by cloning and expressing *tcdR* from its own promoter. Toxin production in the complemented strain was fully recovered (Fig. 1B), whereas the effect on sporulation could be restored only partially (Fig. 2). Unlike toxin gene regulation (where TcdR directly regulates *tcdA* and *tcdB* transcription), sporulation is regulated by multiple transcription factors and alternative RNA polymerase sigma factors (19–21). Sporulation also involves finely tuned spatially and temporally regulated gene expression programs and may not be mimicked exactly in the complemented strain. All of these regulatory mechanisms could result in partial complementation of the sporulation. Another explanation could be that, when the TcdR sigma factor is overexpressed, the availability of RNA core polymerase for other sigma factors needed for sporulation could be limited and that limitation could result in partial complementation of the sporulation phenotype.

10.1128/mSphere.00383-16.2 FIG S1  Construction and characterization of *tcdR* mutant in *C. difficile*. (A) Schematic representation of insertional inactivation of *tcdR* by group II intron. (B) The intron insertion in the *tcdR* coding region was verified by PCR using the intron-specific primer EBS along with gene-specific primers ORG81 and ORG82 in the parent (R20291), in the *tcdR* mutant (R20291::*tcdR*), and in the *tcdR* complemented strain (R20291::*tcdR*+pRG294). The same strategy was followed to verify the *tcdR* mutation in the 630*Δerm* strain. (C) Southern blot analysis of genomic DNA from the WT and *tcdR* mutant strains with a *tcdR*-specific probe. The shift in the hybridization band indicates the integration of the intron within *tcdR* coding region. Download FIG S1, PDF file, 0.2 MB. Copyright © 2017 Girinathan et al. 2017 Girinathan et al. This content is distributed under the terms of the Creative Commons Attribution 4.0 International license.

10.1128/mSphere.00383-16.9 TABLE S1  Oligonucleotides used in the study. Download TABLE S1, DOCX file, 0.1 MB. Copyright © 2017 Girinathan et al. 2017 Girinathan et al. This content is distributed under the terms of the Creative Commons Attribution 4.0 International license.

### Transcriptome analysis of *tcdR* mutant.

The global regulators *ccpA* and *codY* are known to influence both sporulation and toxin production in *C. difficile* (10, 11). We performed quantitative reverse transcription-PCR (qRT-PCR) analysis and found no significant change in their transcript levels in the *tcdR* mutant compared to the WT strain (Fig. S2). Since this initial analysis failed to explain the reasons behind the unexpected phenotype of the *tcdR* mutant, we decided to perform a transcriptome study using RNA-seq analysis. RNAs were prepared from stationary-phase cultures of the *tcdR* mutant (mutant R20291::*tcdR*) and the WT strain (strain R20291) and were subjected to RNA-seq analysis. The data observed for selected genes were confirmed by performing qRT-PCR analysis (Fig. S3 and S4). RNA-seq analysis of the *tcdR* mutant showed that most of the genes were underexpressed and revealed that two major classes of genes were particularly affected, i.e., the PaLoc genes and the sporulation-associated genes (see NCBI GEO accession number GSE85395). However, few genes were upregulated in the *tcdR* mutant. Among those that were overexpressed, we found the *srlR* gene encoding the regulator of glucitol/sorbitol-specific PTS system (CDR20291_0690 to CDR20291_0696). PaLoc genes (*tcdA*, *tcdB*, *tcdR*, and *tcdE*) were downregulated (33-fold, 12-fold, 5-fold, and 3-fold, respectively) in the *tcdR* mutant, as expected. Autoregulation of TcdR and its need for toxin gene transcription were well characterized previously (7, 8, 16). However, no report was available on the TcdR-mediated transcription of *tcdE* in the PaLoc. TcdE is a holin-like protein and was found to mediate toxin release from *C. difficile* cells (22, 23). Our data suggest that TcdR is also needed to initiate *tcdE* transcription in *C. difficile*.

10.1128/mSphere.00383-16.3 FIG S2  Expression analysis of toxin genes and known toxin gene regulators in the *tcdR* mutant. RNA was prepared from R202091 and R20291::*tcdR* strains grown in TY medium for 16 h. qPCR analysis was performed for selected regulator-coding genes. Statistical analysis was performed using the *t* test, and the error bars indicate the standard errors of the means (s.e.m.). **, *P* value < 0.01. Download FIG S2, PDF file, 0.1 MB. Copyright © 2017 Girinathan et al. 2017 Girinathan et al. This content is distributed under the terms of the Creative Commons Attribution 4.0 International license.

### Many sporulation-associated genes were significantly repressed in the *tcdR* mutant.

**FIG 3:**
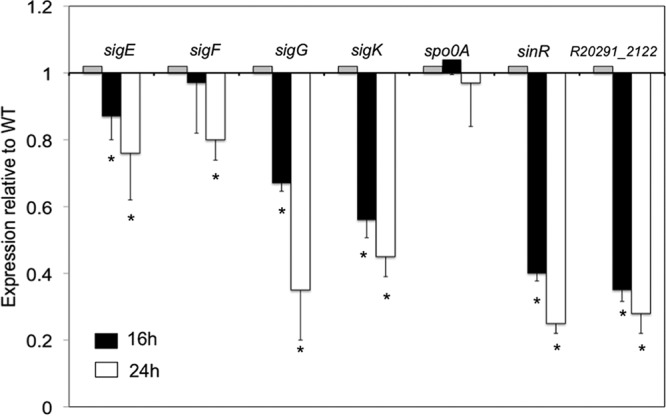
Decreased expression of key sporulation genes in the *tcdR* mutant. Data represent results of qRT-PCR analysis of *sigE*, *sigF*, *sigG*, *sigK*, *spo0A*, *sinR*, and *R20291_2122* expression after 16 and 24 h of *C. difficile* growth in 70:30 sporulation medium. Error bars correspond to the standard errors of the means of results from at least three biological replicates. *, *P* < 0.05 (by two-tailed Student’s *t* test).

In addition to the PaLoc genes, many genes in the sporulation pathways were repressed in the *tcdR* mutant compared to the WT (Table 1). Sporulation is a highly complex cellular process regulated by a cascade of events (20, 21, 24). Spo0A is the master regulator of sporulation, and its transcript levels were unchanged in the *tcdR* mutant as observed in both the RNA-seq and qRT-PCR analyses (Table 1 and Fig. 3). However, we saw that transcripts of specific sporulation sigma factor genes *sigE*, *sigF*, *sigG*, and *sigK* were underexpressed in the *tcdR* mutant (Table 1 and Fig. 3). Even though the levels of transcription of these genes were moderately (1.5-fold to 2-fold) reduced in the *tcdR* mutant compared to the WT strain in the RNA-seq analysis, we observed through qRT-PCR analyses that their transcription levels in the *tcdR* mutant were significantly reduced throughout the time of growth (Fig. 3). RNA-seq analysis also revealed several sporulation genes controlled by *sigE*, *sigG*, and *sigK* to be significantly affected in the *tcdR* mutant (Table 1) (19–21). SigE is a mother cell-specific sigma factor responsible for the transcription of early sporulation-specific genes, and the SigE-regulated genes identified to be affected in *tcdR* mutant included the following: *spoIVA* (stage IV sporulation protein A); *spmBA* (spore maturation proteins B and A); and *sigK*, the second mother cell-specific sigma factor. SigG is the forespore-specific factor that controls the final stages of sporulation. The SigG-regulated genes found to be repressed in *tcdR* mutant included the following: *pdaA* (spore specific deacetylase), *sspA* (small acid-soluble protein), and *spoVAC* and *spoVAD* (stage V sporulation proteins). SigG and SigE activities were previously found to be required for the production of heat-resistant spores (21). The *sigK C. difficile* mutant was able to make heat-resistant spores; however, the level of production was 3 log lower than that seen with the parent strain (21). SigK regulates many genes encoding spore structure proteins that participate in the synthesis of the spore coat and spore exosporium. In fact, we found that many of the SigK-regulated genes such as *cotJBD*, *cotA*, *cotB*, *cotE*, *bclA3*, *and bclA2* as well as the *sleC* and *cdeC* genes were significantly underexpressed in the *tcdR* mutant compared to the WT strain. The downregulation of these genes was confirmed by qRT-PCR analysis (Fig. S3 and S4).

10.1128/mSphere.00383-16.4 FIG S3  Expression analysis of selected sporulation genes during growth in 70:30 medium. RNA was prepared from R202091 and R20291::*tcdR* strains grown in 70:30 medium for 24 h. qRT-PCR analysis was performed for selected sporulation genes. Statistical analysis was performed using the *t* test, and the error bars indicate the standard errors of the means (s.e.m.). *, *P* value < 0.05. Download FIG S3, PDF file, 0.1 MB. Copyright © 2017 Girinathan et al. 2017 Girinathan et al. This content is distributed under the terms of the Creative Commons Attribution 4.0 International license.

10.1128/mSphere.00383-16.5 FIG S4  Expression analysis of selected sporulation genes during growth in TY medium. RNA was prepared from R202091 and R20291::*tcdR* strains that were grown in TY medium for 24 h. qRT-PCR analysis was performed for selected sporulation genes. Statistical analysis was performed using the *t* test, and the error bars indicate the standard errors of the means (s.e.m.). *, *P* value < 0.05. Download FIG S4, PDF file, 0.1 MB. Copyright © 2017 Girinathan et al. 2017 Girinathan et al. This content is distributed under the terms of the Creative Commons Attribution 4.0 International license.

10.1128/mSphere.00383-16.6 FIG S5  TEM analysis of spores from the R20291 and R20291::*tcdR* strains. The *tcdR* mutant spores (*n* = 60) were scored 100% for the presence of ruffled defective exosporium (marked with black arrow) and 98% for the presence of weakly stained core (marked as open triangle). Bar, 100 nm. Download FIG S5, PDF file, 1.5 MB. Copyright © 2017 Girinathan et al. 2017 Girinathan et al. This content is distributed under the terms of the Creative Commons Attribution 4.0 International license.

**TABLE 1:** Differentially expressed sporulation genes in R20291::*tcdR* mutant^a^

Gene ID	Gene name if assigned, known/predicted function	Fold downregulation in mutant (WT/*tcdR* mutant)	Known or predicted sigma factor needed for expression
CDR20291_0124	Cell wall endopeptidase	3.844	SigF
CDR20291_2145	Hypothetical protein	5.993	SigF
CDR20291_2363	*gpr*, germination protease	4.008	SigF
CDR20291_3400	Spore cortex-lytic enzyme	5.652	SigF
CDR20291_3401	*spoIIR*, stage II sporulation protein	4.228	SigF
CDR20291_2530	*sigG*	2.14	SigF
CDR20291_0125	*spoIIID*, stage III sporulation protein D	5.323	SigE
CDR20291_0714	Stage IV sporulation protein	12.140	SigE
CDR20291_1031	*spoIIIAB*, stage III sporulation protein AB	3.600	SigE
CDR20291_1032	*spoIIIAC*, stage III sporulation protein AC	4.031	SigE
CDR20291_1033	*spoIIIAD*, stage III sporulation protein AD	4.458	SigE
CDR20291_1034	*spoIIIAE*, stage III sporulation-related protein	3.733	SigE
CDR20291_2147	*cspBA*, germination-specific protease	4.346	SigE
CDR20291_2513	*spoIVA*, stage IV sporulation protein A	4.773	SigE
CDR20291_3376	*spmB*, spore maturation protein B	4.333	SigE
CDR20291_3377	*spmA*, spore maturation protein A	5.447	SigE
CDR20291_1073	Hypothetical protein	4.563	SigE
CDR20291_0702	*spoVAC*, stage V sporulation protein AC	5.524	SigG
CDR20291_0703	*spoVAD*, stage V sporulation protein AD	5.682	SigG
CDR20291_1130	Small acid-soluble spore protein	4.816	SigG
CDR20291_1131	*dacF*, d-alanyl-d-alanine-carboxypeptidase	5.891	SigG
CDR20291_1529	*sodA*, superoxide dismutase	5.714	SigG
CDR20291_2576	*sspA*, small acid-soluble spore protein A	4.500	SigG
CDR20291_2802	*spoVFB*, dipicolinate synthase subunit B	3.914	SigG
CDR20291_3080	Small acid-soluble spore protein	4.107	SigG
CDR20291_3107	*sspB*, small acid-soluble spore protein B	4.690	SigG
CDR20291_0212	Spore coat protein	6.600	SigK
CDR20291_0316	Spore coat assembly asparagine-rich protein	6.101	SigK
CDR20291_0337	Fragment of putative exosporium glycoprotein	12.666	SigK
CDR20291_0522	*cotJB1*, spore-coat protein	8.666	SigK
CDR20291_0523	*cotJC1*, spore-coat protein	6.842	SigK
CDR20291_2290	*cotJB2*, spore-coat protein	5.679	SigK
CDR20291_2291	*cotJC2*, spore-coat protein	5.165	SigK
CDR20291_2803	*dpaA*, dipicolinate synthase subunit A	4.291	SigK
CDR20291_3090	*bclA2*, exosporium glycoprotein	6.302	SigK
CDR20291_3193	*bclA3*, exosporium glycoprotein	12.612	SigK
CDR20291_3466	Cell wall hydrolase	4.631	SigK
CDR20291_0476	*sleC*, spore peptidoglycan hydrolase	5.502	Partly by SigF, SigK
CDR20291_2121	*sinR*	20.5	Unknown
CDR20291_2122	*sinR* like DNA binding protein	27.25	Unknown
CDR20291_0701	sigF*	1.23	SigH
CDR20291_2531	sigE*	1.56	SigH
CDR20291_1052	spo0A*	1.56	SigH
CDR20291_1067B	sigK*	1.78	SigE

a Genes were considered differentially expressed if the fold change was ≥2.0 and their adjusted *P* value is ≤0.05. Expression levels of genes marked with (*) were not statistically significant. ID, identifier.

### *CDR20291_2121* and *CDR20291_2122* (*sin* operon) were repressed in the *tcdR* mutant.

Other than genes involved in sporulation morphology, we also found some regulatory genes potentially involved in sporulation to be affected in the *tcdR* mutant. In fact, the transcript levels of *CDR20291_2121* (coding for a SinR-like protein of *Bacillus subtilis*) and *CDR20291_2122* (coding for a DNA binding protein) genes were nearly 20-fold lower in the *tcdR* mutant than in the WT strain (Table 1). This result was confirmed by qRT-PCR (Fig. 3). In *B. subtilis*, SinR is encoded within the* sin* locus carrying both *sinI* and *sinR* genes. In *B. subtilis*, SinR forms tetramers, which repress *spo0A* transcription, although SinI is an inhibitor of SinR (25). If SinR functions similarly in *C. difficile*, a decrease in SinR activity should lead to an increase of sporulation. However, we observed decreased sporulation in the *tcdR* mutant (Fig. 2), suggesting that the products of the *sin* locus must function differently in *C. difficile*.

### Spores derived from the *tcdR* mutant have increased heat sensitivity.

**FIG 4:**
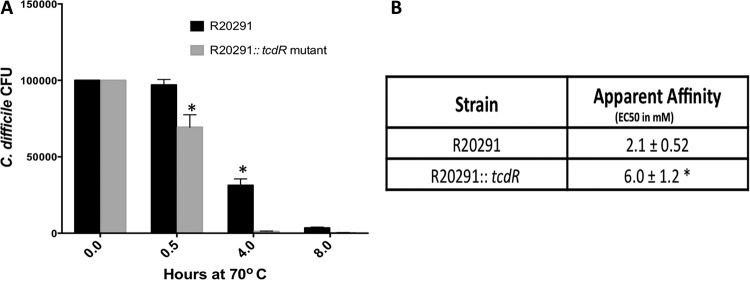
(A) The *tcdR* mutant affects spore germination. Heat resistance of spores of *C. difficile* strain R20291 and its *tcdR* mutant derivatives was measured by heat-treating aliquots at 70°C for 0.5 h, 4 h, and 8 h. The surviving spores were enumerated as described in Materials and Methods. The data represent the averages of the results of three independent experiments, and error bars represent standard errors of the means. Asterisks (*) indicate statistical difference at a *P* value of <0.05. (B) Apparent affinity of taurocholate for *C. difficile* spores. EC_50_s were individually calculated from three independent germination experiments and are reported as averages with standard errors of the means. A Student’s *t* test was performed, and that asterisk indicates that the calculated *P* value is <0.05.

To compare the levels of heat sensitivity of spores between the WT and the *tcdR* mutant strains, we incubated purified spores at 70°C for 0.5 h, 4 h, and 8 h. When we monitored cell viability using the heat-treated spores, we found that spores from the *tcdR* mutant lost most of their viability upon 4 h of heat treatment and that they were nearly 10-fold more sensitive to heat than the WT spores (Fig. 4A). This could have been due to the decreased expression of both *sigG* and *sigE* in the *tcdR* mutant as observed in our transcriptional analysis; their activities are known to be involved in the formation of heat resistance of spores (21). In addition, the lower expression of many of the spore structure proteins (including *cdeC*) in the *tcdR* mutant can also explain the heat sensitivity of these spores.

### Increased taurocholate was required by *tcdR* mutant spores for germination.

To test if the lower transcription of sporulation-associated genes observed in the* tcdR* mutant (Table 1) affects the ability of *C. difficile* spores to germinate, we determined the apparent interaction of spores with taurocholic acid (Fig. 4B). *C. difficile* spores were suspended in rich medium alone or supplemented with increasing concentrations of the germinant taurocholate. The kinetics of spore germination were followed by measuring the rate of the decrease in optical density at 600 nm (OD_600_) as the spores germinated (see Materials and Methods). Though not traditional enzyme kinetics, this assay allows us to understand how spores interact with the taurocholate germinant. *C. difficile* R20291 spores display a 50% effective concentration (EC_50_) of 2.1 mM (similar to what has been previously reported for other strains) (26–28). However, the *tcdR* mutant spores display an EC_50_ of 6.0 mM, corresponding to a 3-fold reduction (<0.05 *P* value) in TA affinity. These results support the overall observation that spore-associated functions were affected when *tcdR* was inactivated in strain R20291.

### Exosporium assembly was affected in the *tcdR* mutant.

**FIG 5:**
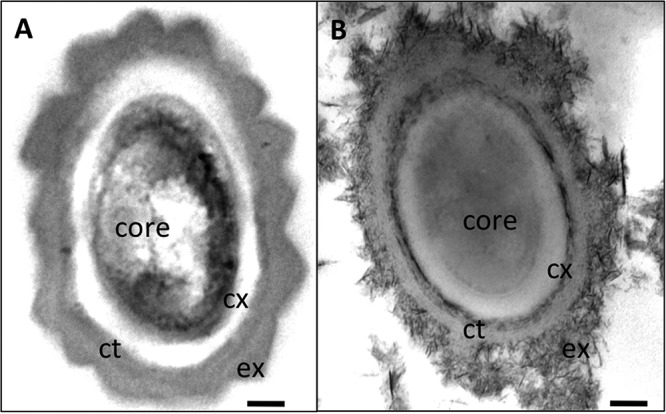
Transmission electron microscopic analysis of *C. difficile* spores. The images show thin sections of spores from the WT R20291 strain (A) and the R20291::*tcdR* mutant (B). Abbreviations: ex, exosporium; ct, coat; co, core; cx, cortex. Bar, 100 nm.

Spores of the R20291::*tcdR* mutant were compared to those of the WT using electron microscopy to assess any effect on gross spore morphology. Samples were viewed as embedded thin sections, and the analysis revealed that *tcdR* mutant spores had a defect in their exosporium assembly (Fig. 5B and S5). The spore core of the *tcdR* mutant was stained weakly compared to the core of the WT spores, and darkly stained particulate materials were present over the spore coat and throughout these preparations. Weaker exosporium in the *tcdR* mutant spores could have made them susceptible to structural changes during chemical fixation procedures, resulting in these darker particles around the spores. In contrast, most of the R20291 WT spore had an intact exosporium that fully enclosed the spore coat (Fig. 5A) and was devoid of the darker debris observed in the *tcdR* mutant spores. This observation suggests that the *tcdR* mutation in R20291 affects the spore structure, with a profound effect on its exosporium assembly.

### The effect of *tcdR* on sporulation is strain specific.

**FIG 6:**
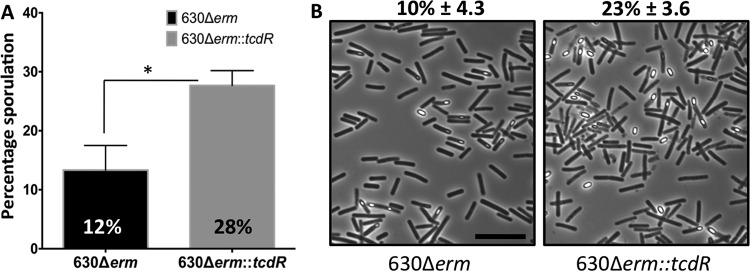
Effect of *tcdR* on sporulation is strain specific. (A) Sporulation frequency (CFU per milliliter of ethanol-resistant spores) of 630Δ*erm* and 630Δ*erm*::*tcdR* strains grown for 24 h in 70:30 sporulation medium. The error bars correspond to standard deviations of results from at least three biological replicates. The asterisk (*) indicates a *P* value of <0.05 (by two-tailed Student’s *t* test). (B) Phase-contrast microscopy of paraformaldehyde-fixed 630Δ*erm* and 630Δ*erm*::*tcdR* strains grown for 48 h in a 70:30 sporulation plate. At least three independent experiments were performed to calculate percent sporulation (± standard errors of the means).

Previous studies have shown that mutations in a specific gene can result in different phenotypes in different *C. difficile* strain backgrounds (29, 30). To understand whether the effect of TcdR is strain dependent, we created a* tcdR* mutant in the 630Δ*erm* strain using a ClosTron system. Toxin production in the 630Δ*erm*::*tcdR* mutant was severely downregulated as observed in strain R20291::*tcdR* (Fig. S6). But unlike the results seen with the R20291::*tcdR* strain, the sporulation efficiency of the 630Δ*erm*::*tcdR* strain was nearly 2-fold greater than that of its WT strain (Fig. 6). The similar opposing phenotype was previously reported for the *spo0A* mutants of strain R20291 versus the 630Δ*erm* mutant, which also affects the toxin production (29, 30). Though the *spo0A* mutation resulted in increased toxin production in the R20291 strain, it resulted in reduced toxin production in the 630Δ*erm* background. Even though the R20291 and 630 strains share 3,247 core genes, their genomes are significantly different from one another (31), whereas there are 47 coding sequences unique in R20291 compared to the 630 strain and 505 coding sequences unique in 630 compared to the R20291 strain (31). Therefore, the difference that we observed in these two strains concerning the impact of the *tcdR* mutation on sporulation might be related to the presence or absence of any of these unique genes. Even though we do not know the exact reason for these differences, these observations suggest that the *C. difficile* genome is dynamic and that its regulatory networks are fluid in nature.

10.1128/mSphere.00383-16.7 FIG S6  Toxin ELISA for 630Δ*erm* and 630Δ*erm*::*tcdR* strains. TcdA and TcdB expression levels in cytosolic fractions of *C. difficile* strains grown for 10 h in TY medium were quantified using ELISA. Data are from results of an experiment representative of three independent assays. Error bars correspond to the standard errors of the means of results from at least three biological replicates. The asterisks (***) in panel B indicate statistical difference at *P* < 0.001, estimated by Student’s *t* test. Download FIG S6, PDF file, 0.04 MB. Copyright © 2017 Girinathan et al. 2017 Girinathan et al. This content is distributed under the terms of the Creative Commons Attribution 4.0 International license.

## DISCUSSION

TcdR-mediated toxin gene regulation is well studied in *C. difficile* (7, 8, 16). The aim of this study was to understand whether TcdR could influence cellular processes other than toxin production. To investigate this issue, we created a *tcdR* mutant in the R20291 strain and performed several phenotypic assays. As expected, the *tcdR* mutant strain either produced no toxins or produced toxins at levels that were not detectable. Surprisingly, we also observed that the level of spores produced by the mutant was significantly reduced compared to the level seen with wild type.

The link between toxin production and sporulation in *C. difficile* has always been suggested but has not been well studied. For example, in *C. difficile* R20291, a mutation in *spo0A*, the master regulator of sporulation, resulted in changes in toxin production (32). More recently, Edwards et al. reported that inactivation of CD3688 (*rstA*) in *C. difficile* strain 630 affects sporulation, toxin production, and motility (33). Moreover, it has been shown that the global regulators CodY and CcpA regulate toxin production along with sporulation (10, 12, 34). Thus, if the *tcdR* mutation affects *codY*, *ccpA*, or *spo0A* expression, both toxin production and sporulation could be influenced. When we measured transcript levels of these genes by qRT-PCR, we found no change in their levels in the *tcdR* mutant compared to the WT (see Fig. S2 in the supplemental material). However, the genome-wide transcriptome analyses of the *tcdR* mutant confirmed that many sporulation genes were affected.

Nearly 50% of the sporulation genes downregulated in the *tcdR* mutant are known (or predicted) to be under the control of SigE and SigK for their transcription (Table 1) (19–21, 35, 36). Among the downregulated SigE-dependent genes, we found *sigK*, whose presence could explain the transcriptional decrease in the levels of several SigK target genes in the *tcdR* mutant. The RNA-seq analyses of the *tcdR* mutant showed that transcription of *spoIIR* and *spoIIID* genes was reduced (Table 1). SpoIIR is essential for the activation of SigE (19, 35), and *spoIIID*, encoding a transcriptional regulator, is involved in the transcription of *sigK* (36). In *C. difficile*, as in *B. subtilis*, SigE is activated by proteolytic cleavage of the SigE precursor form (pro-SigE) (20). In *B. subtilis*, the enzyme SpoIIGA, which is responsible for pro-SigE processing, is coexpressed with *sigE* and is activated only when the mother cell and forespore compartments are formed (37–39). The trigger for SpoIIGA activation is the SpoIIR signal protein that is synthesized in the newly formed forespore and whose presence is communicated to the mother cell (40, 41). In *B. subtilis*, *spoIIR* is regulated by SigF, whereas in *C. difficile*, partial SigE processing is observed in *sigF* mutants, suggesting a lower level expression of *spoIIR* in the absence of SigF (19, 20). If the expression of *spoIIR* in a *sigF* mutant is influenced by TcdR, this could explain the partial processing of SigE in *sigF* mutants. Thus, a reduced abundance of *spoIIR* in the *tcdR* mutant could lead to low levels of activated SpoIIGA and part of pro-SigE would remain unprocessed and inactive. If so, this would also result in a decrease of *spoIIID* levels as observed in the transcriptome (Table 1); therefore, little or no transcription of *sigK* would occur, resulting in poor spore maturation.

Most of the genes identified as affected in the *tcdR* mutant code for proteins that are part of the spore proteome (20, 32, 42) and are involved in spore structure and germination. To determine whether *tcdR* mutant spore properties are different from those of the WT spores, we performed heat sensitivity and germination assays using purified spores. TcdR mutant spores were 10 times more heat-sensitive than WT spores (Fig. 4A). Accordingly, transcriptome analysis showed that several exosporium and coat protein coding genes were underexpressed in the *tcdR* mutant. A recent study on the *C. difficile* exosporium protein BclA3 demonstrated its role in spore heat resistance (43). The authors found that BclA3 is glycosylated by a glycosyltransferase encoded by the adjacent gene (CD3350) within the same operon whose mutation resulted in unglycosylated BclA3. They showed that spores from this mutant were highly susceptible to heat treatment compared to the WT spores (43). The same heat susceptibility was observed with the exosporium protein CdeC, which is present only in *C. difficile* and is needed for the assembly of exosporium (44). Also, *C. difficile spoVAC* and *dpaAB* mutants produced heat-sensitive spores (45). The dipicolinate synthase enzyme subunits (SpoVFB and DpaA) are responsible for the production of dipicolinic acid (DPA), which protects spores during heat treatment (46–48). Moreover, proteins encoded in the *spoVA* operon are responsible for transporting DPA from the mother cell to forespores during spore development (49).

All these results are consistent with the transcriptome analysis of the *tcdR* mutant, which showed decreased expression of *bclA3*, *cdeC*, *spoVAC*, and the DPA synthase coding operon. This probably results in the production of spores with weaker exosporium that must be more sensitive to heat treatment than the WT strain (Fig. 4A). Transmission electron microscopy (TEM) analysis of the *tcdR* mutant spores confirmed this speculation, where the exosporium was found to be defective and weakly assembled (Fig. 5).

Germination of bacterial spores is induced when the germinant receptors (GR) sense germinants and subsequently trigger the release of spore core DPA (46). The release of DPA from the spore core leads to the activation of cortex hydrolases that degrade the peptidoglycan (PG) cortex layer, which then allows core hydration. In *C. difficile*, CspC is the bile salt-sensing germinant receptor and is necessary for the release of DPA from spores (26). SleC is the spore cortex lytic enzyme, and its activation depends on CspC (through CspB-mediated cleavage of the prodomain to generate active SleC) (26, 50–52). A mutation in *sleC* was previously reported to affect germination in *C. difficile* (51, 53). Thus, lower transcription of *sleC* in *tcdR* mutant (Table 1) suggested that *tcdR* mutant spores could have inefficient germination. In agreement, we have shown that the TA affinity of *C. difficile tcdR* spores is low compared to that of the WT spores (Fig. 4B), indicating that germination is significantly reduced.

Several studies have previously identified the TTTACA sequence as the −35 region of the TcdR-dependent promoters (7, 8). To test whether some of the downregulated sporulation genes in the *tcdR* mutant can be directly controlled by TcdR, we looked for the presence of this consensus sequence in the promoter regions of these genes (Table 1). In fact, we found 9 genes/operons carrying the sequence in the −35 region of the TcdR-dependent promoters. These genes include *bclA2*, *bclA3*, *cotJBD*, *spoVFB*, *cotA*, *cotB*, *cotE*, *dpaA*, and *sin*. To test if any of these genes are directly controlled by TcdR, we constructed transcriptional fusions between the promoter of the *bclA2* and *bclA3* genes and the *Escherichia coli* β-glucuronidase (*gusA*) gene that we introduced in a *gus*-negative *E. coli* strain expressing or not expressing TcdR as we did previously (54). Compared to the control strains, we did not see any TcdR-mediated transcription of *bclA2* or *bclA3* promoters, indicating that TcdR is not a direct regulator of these genes (Fig. S7). However, we cannot exclude for these genes the possibility that TcdR may act together with a specific regulator present in the R20291 strain.

10.1128/mSphere.00383-16.8 FIG S7  Beta-glucuronidase activity of *bclA2* promoter-*gusA* and *bclA3* promoter-*gusA* fusions in the presence or absence of TcdR. *E. coli* strains carrying promoter fusion plasmids (*pbclA2-gusA* and *pbclA3-gusA*) along with a TcdR-expressing plasmid (pRGL312) or the vector (pET16b) were grown for 6 h, and TcdR expression was induced with 1 mM IPTG (isopropyl-β-d-thiogalactopyranoside) for 4 h at 37°C. Bacterial cultures were harvested and were assayed for beta-glucuronidase activity (quantified in Miller units) as described previously (18). The values represent the means of the results from three independent experiments. Statistical analysis was performed using the *t* test, and the error bars indicate standard errors of the means. The asterisks (**) indicate statistical difference at *P* < 0.005, estimated by Student’s *t* test. Download FIG S7, PDF file, 0.1 MB. Copyright © 2017 Girinathan et al. 2017 Girinathan et al. This content is distributed under the terms of the Creative Commons Attribution 4.0 International license.

Finally, the effect of TcdR on sporulation could be indirect. TcdR is an alternate sigma factor, and its presence or absence could influence the availability of the RNA polymerase core enzyme for other sigma factors in the cell, which in turn can influence the gene expression pattern. Thus, the absence of TcdR in the R20291::*tcdR* strain, increasing the availability of RNA polymerase core enzyme to other sigma factors, could indirectly affect those involved in the sporulation process. On the other hand, there may be common regulators that connect toxin gene regulation with the sporulation pathway in *C. difficile* that could be affected by the *tcdR* mutation. Previous studies have identified several regulators in *C. difficile* regulating toxin production along with sporulation, which strongly suggests that these two pathways were linked (10, 12, 33, 34, 55).

In the past decade, large *C. difficile* outbreaks, with higher relapse rates and increased mortality rates, were reported throughout the world and were attributed to *C. difficile* strains belonging to ribotype 027. Strain R20291 used in this study is a ribotype 027 isolate (56). Genetic and phenotypic features of this ribotype hint that the strains grouped as 027 ribotypes are different from other *C. difficile* strains (31). Recently, Lyon et al. reported that CdtR, a regulator in the binary toxin locus CdtLoc, could regulate toxin production only in 027 ribotypes and not in others (57). The authors of that study proposed that CdtR could be regulating toxin production by regulating the TcdR through a yet-to-be-identified intermediary regulator in the 027 ribotype. It has been previously proposed that the ability to regulate toxin production in response to various environmental cues with various regulatory responses may be different for 027 ribotypes in comparison to other *C. difficile* ribotypes (31). Results from subsequent studies are in agreement with this proposal. For example, a mutation in the highly conserved *codY* gene results in different phenotypes from 027 ribotypes and other ribotypes. The *codY* mutation results in a hypersporulation phenotype in a 027 ribotype (UK1 strain) and produces only a moderate effect on the sporulation in an 012 ribotype (630 strain) (34). It is also worth noting that *sin* locus expression levels were different in *codY* mutants in these two different *C. difficile* backgrounds (34). Similarly, a mutation in *spo0A* resulted in increased toxin production only in the 027 ribotype and not in the 012 ribotype (29, 30). In the current study, we observed the positive influence of TcdR on sporulation only in R20291 of the 027 ribotype and not in strain 630 of ribotype 012. Even though those previous studies, along with our observations, suggested that ribotype 027 has unique gene regulatory networks that differ from those of other *C. difficile* strains, variations may be present in strains within the 027 ribotype. Detailed study is needed to check whether the gene regulatory networks of the toxin synthesis and sporulation pathway are connected in all known ribotype 027 strains. In such a case, the ability to synchronize the toxin production and the sporulation can provide the selective advantage to ribotype 027 isolates to enable them to be more successful, with increased virulence and high transmission abilities. Deciphering the connections between toxins and the sporulation regulatory network could lead to the discovery of other novel regulators and pathways that can be targeted for the development of new therapeutics to manage *C. difficile* infections. Any treatment that leads to inhibition of toxin production and spore formation in patients with *C. difficile* infection can potentially lower the severity of the disease in addition to the transmission and recurrence of infection through dissemination of the spores.

## MATERIALS AND METHODS

### Bacterial strains and growth conditions.

*Clostridium difficile* strains (Table 2) were grown anaerobically in TY agar (tryptose, yeast extract) or 70:30 medium (58) as described previously (15, 54). Cefoxitin (Cef; 25 µg/ml), thiamphenicol (Thio; 15 µg/ml), and lincomycin (Lin; 15 µg/ml) were added to *C. difficile* cultures whenever necessary. *Escherichia coli* strains were grown in (LB) broth. *E. coli* strain S17-1 (59), used for conjugation, was supplemented with ampicillin (100 µg/ml) or chloramphenicol (25 µg/ml) when indicated and cultured aerobically in LB broth.

**TABLE 2:** Bacterial strains and plasmids used in this study

Strain or plasmid used	Description	Reference or source
Strains		
*C. difficile* R20291	NAP1/027 ribotype	31
*C. difficile* R20291::*tcdR*	R20291with intron insertion in *tcdR* gene	This study
*C. difficile 630Δerm*	Erm^′^ derivative of strain 630	63
*C. difficile 630Δerm*::*tcdR*	*630Δerm* with intron insertion in *tcdR* gene	This study
*E. coli* DH5α	*endA1 recA1 deoR hsdR17* (*r*_K_^−^ *m*_K_^+^)	NEB laboratories
*E. coli* S17-1	Strain with integrated RP4 conjugation transfer function for conjugation between *E. coli* and *C. difficile*	59
*E. coli* GM241(DE3)	*gusA* mutant lysogenized with DE3 phage and host for *gusA* reporter plasmids	54
Plasmids		
pMTL007-CE5	ClosTron plasmid	18
pMTL007-CE5::*tcdR*-141	pMTL007-CE5 carrying *tcdR*-specific intron	This study
pRPF185	*C. difficile* shuttle vector	64
pRGL294	pRPF185 with *tcdR* expressed from its own promoter	This study
pACYC184	*E. coli* cloning vector; compatible with pET16B	Neb
pACYC515	pACYC184 vector carrying *gusA* gene under the control of the *tcdR* promoter	54
pET16b	*E. coli* expression vector	Novagen
pRGL312	pET16B with *tcdR*	This study
pRGL320	pACYC184 vector carrying *gusA* gene under the control of the *bclA2* promoter	This study
pRGL321	pACYC184 vector carrying *gusA* gene under the control of the *bclA3* promoter	This study
*C. difficile* R20291::*tcdR* + pRGL294	R20291::*tcdR* complemented with *tcdR*	This study
*C. difficile* R20291::*tcdR* + pRPF185	R20291::*tcdR* with vector control	This study

### Construction of a* tcdR* mutant.

A *tcdR* mutation was constructed in a *C. difficile* strain using a ClosTron gene knockout system (18). The group II intron insertion site in the antisense orientation between nucleotides 141 and 142 of the *tcdR* ORF was selected using the Perutka algorithm, a Web-based design tool available at http://www.clostron.com. The designed retargeted intron was cloned into pMTL007-CE5, and the resulting plasmid, pMTL007-CE5::Cdi-*tcdR*-141a, was transferred into R20291 by conjugation as described previously (15, 22). The selection of thiamphenicol-resistant transconjugants in 15 µg·ml^−1^ lincomycin plates confers potential *Lactococcus lactis ltrB* (Ll.ltrB) insertions within the target *tcdR* gene in the chromosome of R20291. The presence of a putative *tcdR* mutant was identified by PCR using *tcdR*-specific primers (Table S1) in combination with the EBSu universal and ERM primers. Specific single integration of the group II intron into the genome was verified by Southern blotting using a (^32^P)dATP-radiolabeled probe specific for the *tcdR* gene as described previously (15, 22). Complementation of the *C. difficile* R20291::*tcdR* mutant is described in Text S1 in the supplemental materials.

10.1128/mSphere.00383-16.1 TEXT S1  Supplemental methods. I. Spore preparation. II. Toxin ELISA. III. Complementation of *tcdR* mutant. IV. RNA-seq analysis. V. Quantitative reverse transcriptase PCR. Download TEXT S1, DOCX file, 0.03 MB. Copyright © 2017 Girinathan et al. 2017 Girinathan et al. This content is distributed under the terms of the Creative Commons Attribution 4.0 International license.

### Toxin assays.

Cultures of R20291 and the R20291::*tcdR* mutant were centrifuged after 10 h in TY medium, and toxin ELISAs were performed as described previously (15). Details are presented in Text S1.

### Sporulation assay (microscopic analysis).

*C. difficile* cultures were grown overnight in TY medium supplemented with 0.1% taurocholate to induce germination of any spores that were present. Cells were then diluted in TY medium to an OD_600_ of 0.5, and then 100 µl was spread on 70:30 sporulation agar (58). Plates were incubated at 37°C and monitored for the production of spores. Cells were harvested from the plates after 24 h and were suspended in TY medium for phase-contrast microscopy as described previously (58). At least four fields per strain were obtained, and the numbers of spores and vegetative cells were counted to calculate the percentage of spores based on the total numbers of spores and vegetative cells. Experiments were performed at least three independent times.

### Sporulation assay (ethanol resistance method).

*C. difficile* strains were inoculated into and grown on 70:30 sporulation agar as described above. After 24 h of growth, cells were scraped from the plates and suspended in 70:30 sporulation liquid medium to an OD_600_ of 1.0. Cells were immediately serially diluted and plated onto TY agar–0.1% taurocholate to enumerate viable vegetative cells and spores. A 0.5-ml aliquot of the culture was removed from the chamber, mixed with 0.5 ml of 95% ethanol, subjected to vortex mixing, and incubated at room temperature for 15 min. Ethanol-treated cells were serially diluted in 1× phosphate-buffered saline (PBS), returned to the anaerobic chamber, and plated onto TY agar–0.1% taurocholate plates to enumerate spores. After 24 h of growth, CFU were enumerated, and percent sporulation was calculated as the number of ethanol-resistant spores divided by the total number of viable cells (vegetative cells and spores).

### Spore preparation.

Spores were generated and purified as previously described (26, 27). Details are presented in Text S1.

### RNA-seq analysis and quantitative reverse transcription-PCR (qRT-PCR).

RNA-seq analysis was performed at the DNA Core Facility at the University of Missouri, and the data were analyzed using methods described previously (60–62). Details of the RNA-seq analysis and the qRT-PCR (19, 55) are provided in Text S1.

### Germination.

Purified *C. difficile* spores were heat activated at 65°C for 30 min and then placed on ice. Ten microliters of the heat-activated spores was added to reach a final optical density at 600 nm (OD_600_) of 0.5 in 990 µl of BHIS medium (brain heart infusion [Difco] supplemented with 5 g/liter yeast extract and 0.1% l-cysteine) alone or supplemented with a 2, 5, 10, 20, or 50 mM concentration of taurocholic acid (TA). Germination was monitored at 600 nm for 30 min in a PerkinElmer (Waltham, MA) Lambda25 UV/Vis spectrophotometer. The data points at OD_600_ (*T*_x_) were normalized to the starting OD_600_ value (*T*0). The germination rates and the 50% effective concentration (EC_50_) were calculated using the slopes of the linear portions of the germination plots as described previously (26, 28). The EC_50_ is the concentration of germinant needed to reach 50% of the maximum germination rate. EC_50_s were individually calculated from each germination experiment and are reported as averages with standard errors of the means.

### Spore heat resistance.

Purified spores (nearly 1 × 10^5^) prepared as described above were resuspended in 500 µl of water and incubated at 70°C. Samples were removed at 0.5 h, 4 h, and 8 h, serially diluted in PBS, plated onto TY agar plates with 0.1% taurocholate, and grown anaerobically for 48 h before counting was performed (44, 45). As a control for non-heat-treated spores, an aliquot was plated onto TY agar–0.1% taurocholate plates prior to the experiment and colonies were counted as described above.

### Transmission electron microscopy.

All steps in sample preparation were performed at room temperature using pelleted spores in a 1.5-ml microcentifuge tube, and solutions were prepared in 1× PBS unless indicated otherwise. For transmission electron microscopy, spores (10^10^) were fixed for 2 h in a solution of 2% glutaraldehyde–2% paraformaldehyde. The spores were thoroughly rinsed three times in 1× PBS (for 5 min each time) and postfixed with 1% osmium tetroxide with constant rotation for 1 to 2 h. The samples were then washed thrice with 1× PBS (for 5 min each time) and stained *en bloc* with 2% aqueous uranyl acetate for 1 h under light-protected conditions and then washed three times (for 5 min each time) with distilled water. The spores were further dehydrated in a graded 50% (vol/vol)-to-95% (vol/vol) acetone series for 5 min and left in 100% acetone overnight. Infiltration was carried out in graded acetone/EMBED 812/araldite resin (Electron Microscopy Sciences) at ratios of 1:1 and 1:2 for 10 min each time at room temperature with constant rotation and incubated in 100% resin overnight. The resin was cured at 60°C for 24 to 48 h, and thin sections (silver to gold color) were cut and absorbed onto on 200-mesh copper grids. Sections were examined with a transmission electron microscope (CM100; FEI Company) at 100 kV, and images were captured using a side-mounted Hamamatsu digital camera (model C8484) with AMT image capture software version 602.591n.

### Accession number(s).

Sequence data have been deposited in the NCBI GEO database under accession number GSE85395.
