# Supplementary figures and images for: Effect of tcdR Mutation on Sporulation in the Epidemic Clostridium difficile Strain R20291
Source: mSphere. 2017 Feb 15;2(1):e00383-16. doi: 10.1128/mSphere.00383-16 (PMC5311115; doi:10.1128/mSphere.00383-16)

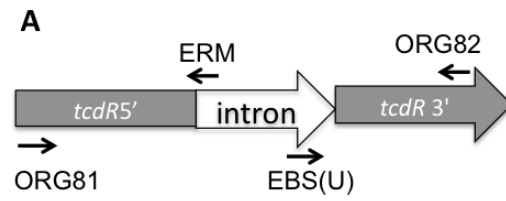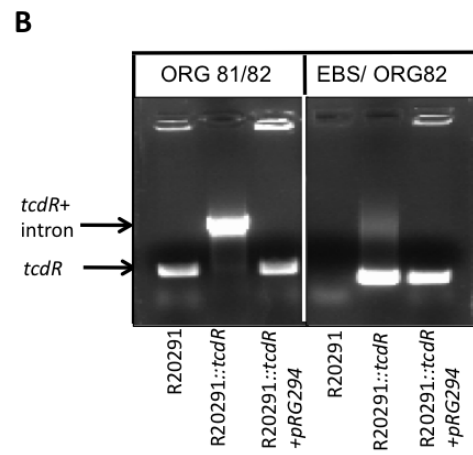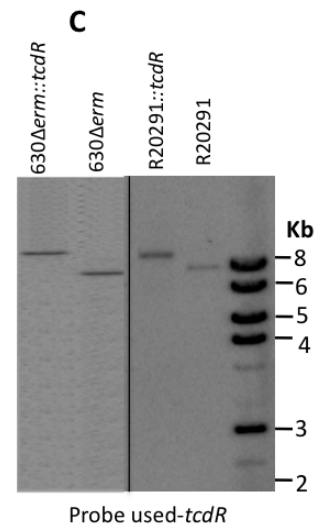

Supplement: FIG S1 [file sph002172235sf3.pdf]

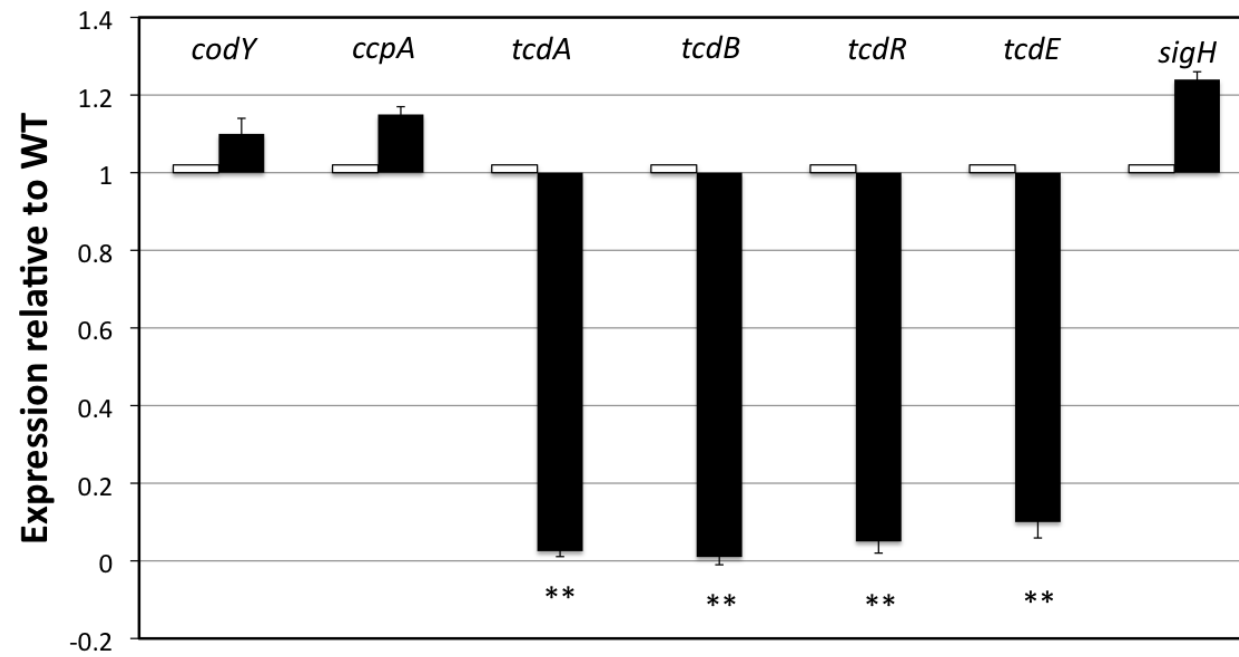

Supplement: FIG S2 [file sph002172235sf4.pdf]

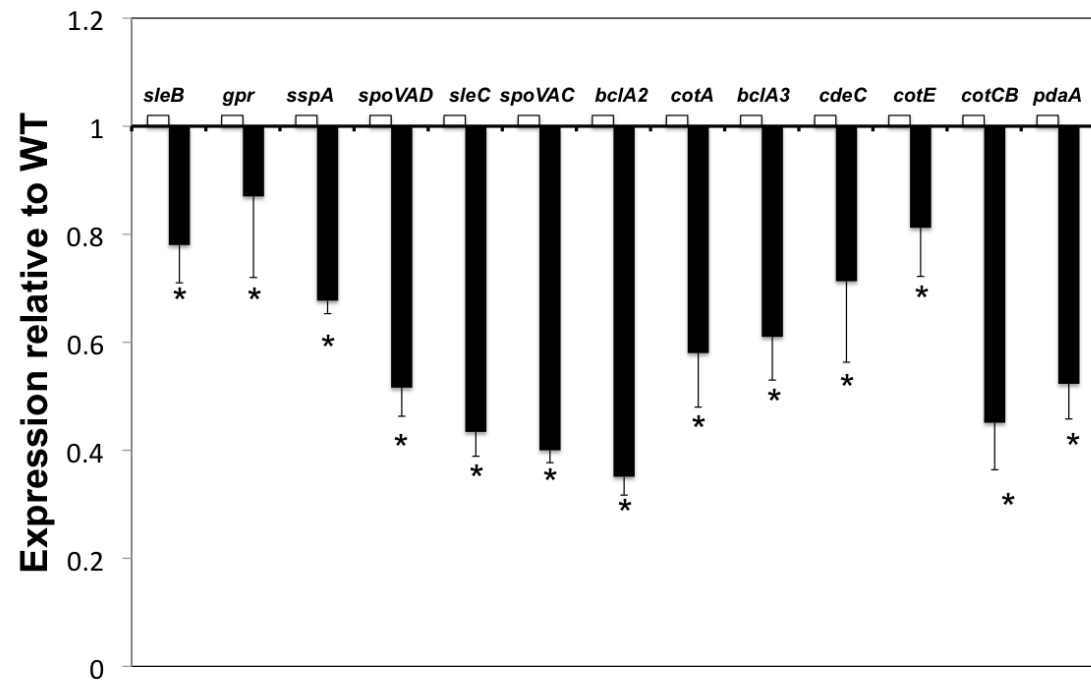

Supplement: FIG S3 [file sph002172235sf5.pdf]

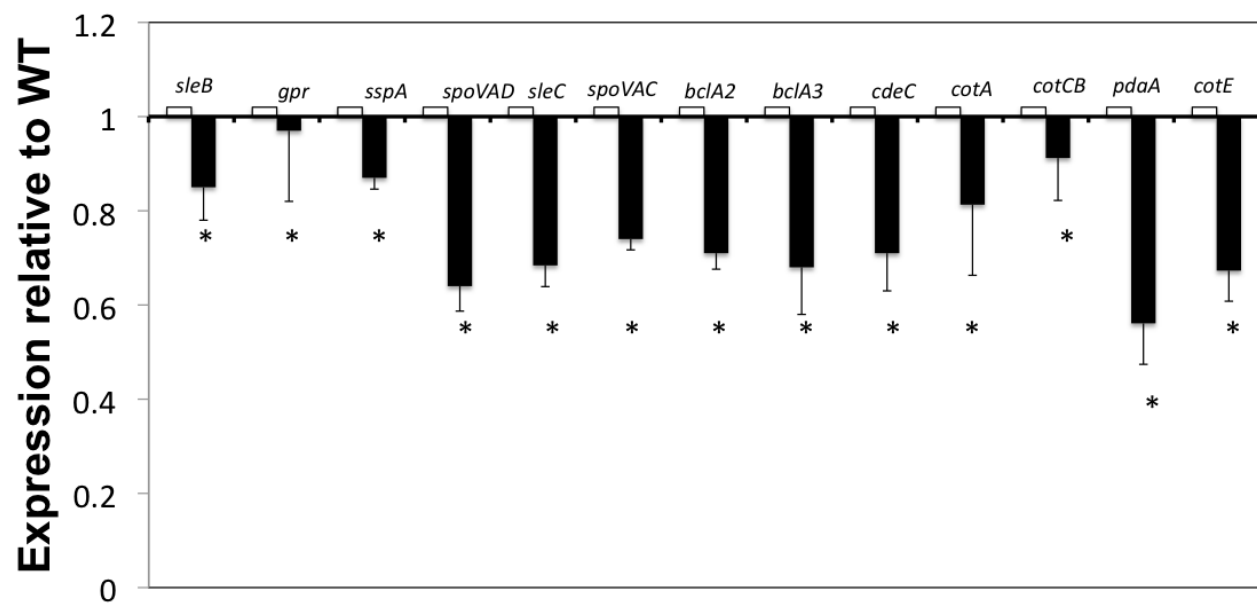

Supplement: FIG S4 [file sph002172235sf6.pdf]

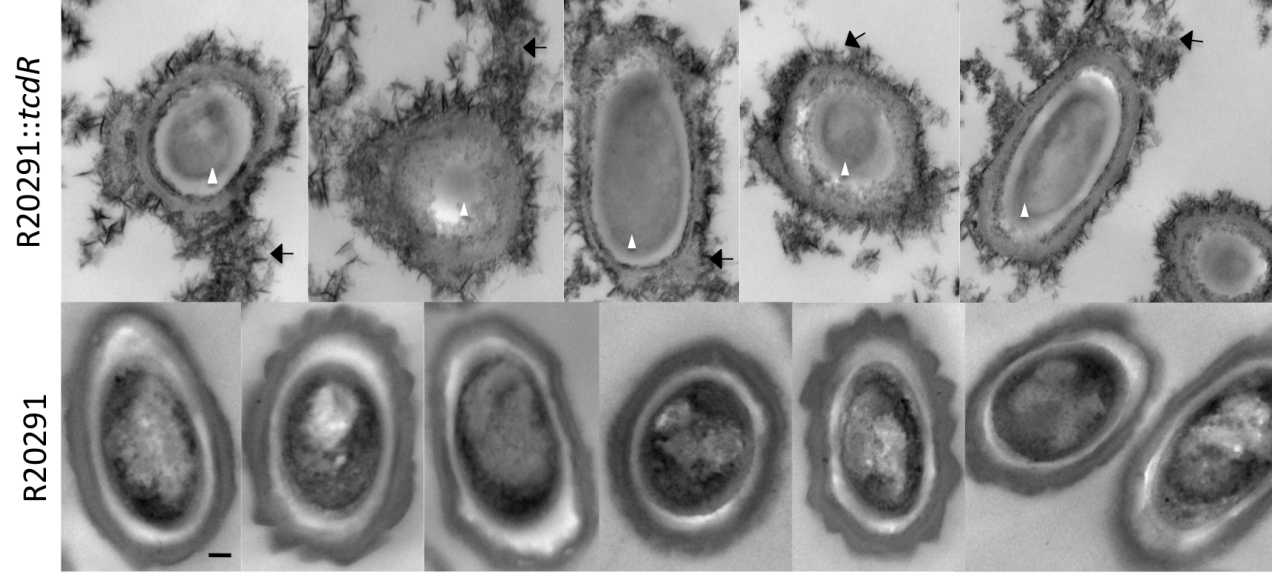

Supplement: FIG S5 [file sph002172235sf7.pdf]

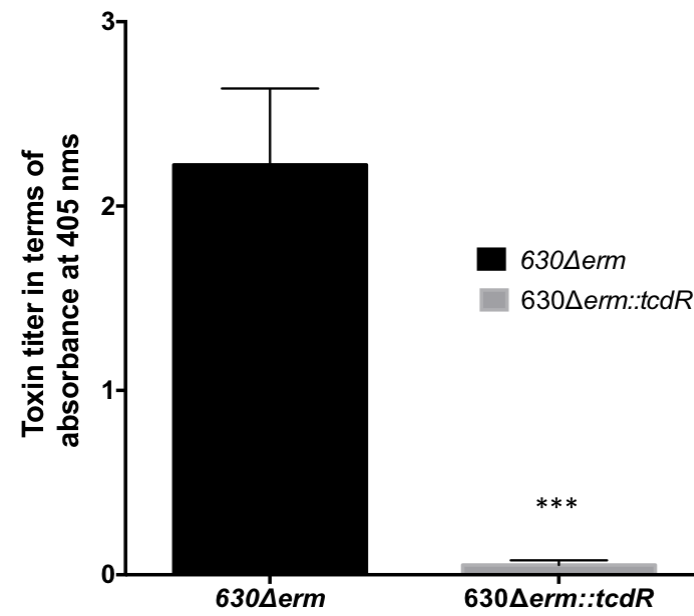

Supplement: FIG S6 [file sph002172235sf8.pdf]

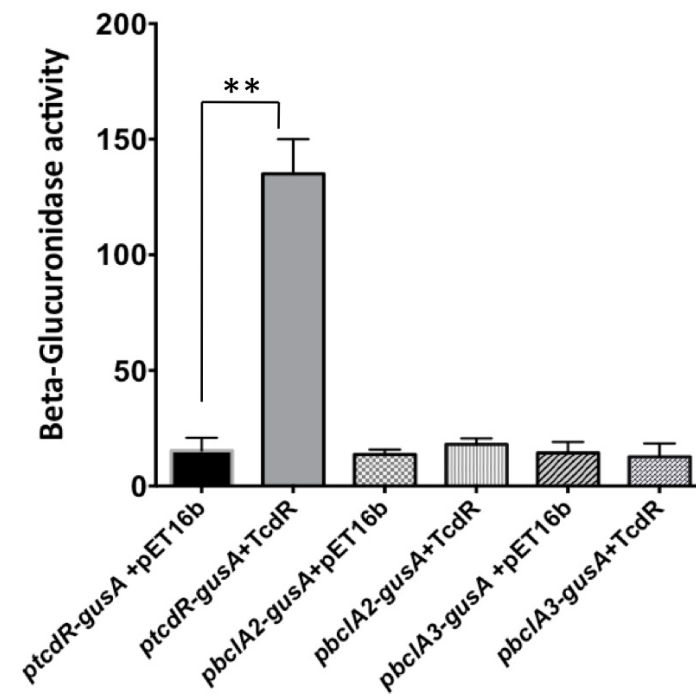

Supplement: FIG S7 [file sph002172235sf9.pdf]
