# Supplementary material for: Effect of tcdR Mutation on Sporulation in the Epidemic Clostridium difficile Strain R20291
Source: mSphere. 2017 Feb 15;2(1):e00383-16. doi: 10.1128/mSphere.00383-16 (PMC5311115; doi:10.1128/mSphere.00383-16)
Supplement: TABLE S1 [file sph002172235st2.docx]

**Effect of *tcdR* mutation on sporulation in the epidemic *Clostridium difficile* R20291 strain**

Brintha P. Girinathan^1^, Marc Monot^2^, Daniel Boyle^1^, Kathleen N. McAllister^3^, Joseph A. Sorg^3^, Bruno Dupuy^2^ and Revathi Govind^1#^

**Table S1**

Oligonucleotides used in this study

| OLIGO Name | Sequence | Description |
| --- | --- | --- |
| EBS universal | CGAAATTAGAAACTTGCGTTCAGTAAAC | Intron specific |
| *tcdR*-IBS | AAAAAAGCTTATAATTATCCTTAGAAACCGATTTAGTGCGCCCAGATAGGGTG | *tcdR* intron retarget |
| *tcdR*-EBS1d | CAGATTGTACAAATGTGGTGATAACAGATAAGTCGATTTAATTAACTTACCTTTCTTTGT | *tcdR* intron retarget |
| *tcdR*-EBS2 | TGAACGCAAGTTTCTAATTTCGATTGTTTCTCGATAGAGGAAAGTGTCT | *tcdR* intron retarget |
| ORG403 | GGTACCCATTTGATTAAAAATAACAAAATATTAAATAATTC | *tcdR* upstream- forward with KpnI |
| ORG81 | ATGCAAAAGTCTTTTTATGAATTAATTGTT | *tcdR coding-Forward* |
| ORG82 | GTTAAAATAATTTTCATAGTCTTTTTTTA | *tcdR* coding-Reverse |
| ORG208 | GAGCTCATATAAGAGAGGATGATTTTATGC | *tcdR coding-Forward* |
| ORG209 | GGATCCTTAATGATGATGATGATGATGCAAGTTAAAATAATTTTC | *tcdR* coding-Reverse with BamHI |
| RG-RT37 | TGACTTTACACTTTCATCTGTTTCTAGC | *sigE* qPCR-Forward |
| RG-RT38 | GGGCAAATATACTTCCTCCTCCAT | *sigE* qPCR-Reverse |
| RG-RT41 | CGCTCCTAACTAGACCTAAATTGC | *sigF* qPCR-Forward |
| RG-RT42 | GGAAGTAACTGTTGCCAGAGAAGA | *sigF* qPCR -Reverse |
| RG-RT49 | CATATGTTGCTAATCGAGTTCCTTTAT | *sigK* qPCR-Forward |
| RG-RT50 | TCAACGGAAGATCAGGATGATTTA | *sigK* qPCR-Reverse |
| RG-RT45 | CAAACTGTTGTCTGGCTTCTTC | *sigG* qPCR-Forward |
| RG-RT46 | GTGGTGTTAATACATCAGAACTTCC | *sigG* qPCR-Reverse |
| RG-RT33 | CATGAAATAGGAGTACCAGCTCA | *spo0A* qPCR-Forward |
| RG-RT34 | CTCCATGCAACCTCTATTGC | *spo0A* qPCR-Reverse |
| RG-RT23 | GAGGAGAGTGGAATTCCTAGTGTAG | *16srRNA* qPCR-Forward |
| RG-RT24 | GGACTACCAGGGTATCTAATCCTGT | *16srRNA* qPCR-Reverse |
| RG-RT25 | AGGCAGGTTTACATCCAACATA | *sinR* qPCR |
| RG-RT26 | AGTGGTATGTCTAAAGCAGTAGC | *sinR* qPCR |
| RG-RT27 | AAAGACTTAAAGAAGAACGGAAAA | CDR20291_2122-Forward |
| RG-RT28 | TTGGATTCTTTTTACCACTTTCG | CDR20291_2122-Reverse |
| sleB-RT | GATATTGTAGAGAACCCCTAATCC | QRT-Forward |
| sleB-RT | GCAAATCCTAAAGCTAAAAATAC | QRT-Reverse |
| Gpr-RT | GGTGTTACTATTAAGTTCTTGTCAT | QRT-Forward |
| Gpr-RT | CTGGTGGAGGTGTTGGCAATACTAG | QRT-Reverse |
| sspA-RT | CTATCTGTTGCTTTTTCCAGCC | QRT-Forward |
| sspA-RT | GTATGAGTAATTATCAACAAGTTG | QRT-Reverse |
| spoVAC-RT | GTAGACCAAATAAGCCCAAAACC | QRT-Forward |
| spoVAC-RT | CAGAACTAGCACCTAGTTTATC | QRT-Reverse |
| spoVAD-RT | gtggcgatttaataaatcaaatag | QRT-Forward |
| spoVAD-RT | cacttcctgctcctgtaactgtcc | QRT-Reverse |
| bclA3-RT | CTGCTGCGTTTGTAAGGTCTATTAC | QRT-Forward |
| bclA3-RT | GAGCAACAGGTCCAACAGGAGCAAC | QRT-Reverse |
| bclA2-RT | GAGTTACTCCATTAAAGTTAG | QRT-Forward |
| bclA2-RT | GGAGTAGCAGGAGCGATAGGACC | QRT-Reverse |
| cdeC-RT | GATGAAATAAATTCAGAAGACATGA | QRT-Forward |
| cdeC-RT | GGCACTGCATTTGATACAGAGAAG | QRT-Reverse |
| sleC-RT | CTGTTCCATAGATACCATCTTC | QRT-Forward |
| sleC-RT | GGGCAGTAAAGACTTAGGTGACC | QRT-Reverse |
| cotCB-RT | ggtacagaggaaatggctcatgttg | QRT-Forward |
| cotCB-RT | cttgtagtaaagtttactccattag | QRT-Reverse |
| cotE-RT | GAATATTGATAAAGCATCATCATATG | QRT-Forward |
| cotE-RT | GCCATAAGAGATGTTATAGGGGATG | QRT-Reverse |
| cotB-RT | GATTTTATCTTACACTGTTCTATTCC | QRT-Forward |
| cotB-RT | GGACCATATTATGATGGAACATGCTC | QRT-Reverse |
| cotA-RT | CTTACCTAGAACTTCAACACCAGTTA | QRT-Forward |
| cotA-RT | CATTGTGTAATCTTAAAGCTGTTGC | QRT-Reverse |
| pdaA-RT | CATCTAATATCTCAGTATTTGTTG | QRT-Forward |
| pdaA-RT | GCGAACAATCTTTAAAATATACACAA | QRT-Reverse |
| pbclA2-F | GGTACCAGATAAGCAATTATATAATTTTGTGGATGCCTTA | *bclA2* promoter-Forward |
| pbclA2-R | TCTAGATAATTAATCCTCCTTTTTTAAAGTTAGAGTATTAC | *bclA2* promoter-Reverse |
| pbclA3-F | GGTACCTAATGAATAGGAATGAATAGGAATGAATAAAGTA | *bclA3* promoter-Forward |
| pbclA3-R | TCTAGAACAACTCCTTTGTCTTCTATATATTGCAGACATAA | *bclA3* promoter-Reverse |
