# Supplementary material for: Effect of tcdR Mutation on Sporulation in the Epidemic Clostridium difficile Strain R20291
Source: mSphere. 2017 Feb 15;2(1):e00383-16. doi: 10.1128/mSphere.00383-16 (PMC5311115; doi:10.1128/mSphere.00383-16)
Supplement: TEXT S1 [file sph002172235s1.docx]

**Effect of *tcdR* mutation on sporulation in the epidemic *Clostridium difficile* R20291 strain**

Brintha P. Girinathan^1^, Marc Monot^2^, Daniel Boyle^1^, Kathleen N. McAllister^3^, Joseph A. Sorg^3^, Bruno Dupuy^2^ and Revathi Govind^1#^

**Supplementary methods: I.** Spore preparation; II. Toxin ELISA, III; Complementation of *tcdR* mutant; IV. RNA seq analysis; V. Quantitative reverse transcriptase PCR.

**I. Spore preparation from *C. difficile* cultures**

*C. difficile* strains were streaked onto 20 plates of BHIS agar medium. After 4 days, the growth from each plate was scraped into 1 ml of sterile water and incubated overnight at 4 °C. The following day, the growth suspension was washed five times with sterile water. In between each wash step, the layer of white cell debris was removed. After washing, the spores/cell debris suspension in water was layered on top of a 60% (w / v) sucrose solution. The gradient was centrifuged in a swinging-bucket rotor at 3,200 X g for 20 minutes. During the centrifugation, the dense spores travel through the sucrose and form a pellet on the bottom while the cell debris is caught in the upper layer. After centrifugation, the solution was removed and the pellet containing the spores was washed five times with sterile water to remove any sucrose. The spores were then suspended in sterile water up to 1 ml. Purified spores were examined under phase contrast microscopy and determined to be >99.9% pure and phase bright.

**II. Toxin ELISA**

Bacterial cells were harvested and were resuspended in 10mM Tris buffer (pH 8.0) containing a protease inhibitor cocktail (Roche, Mannheim, Germany) and sonicated to release the cytosolic contents. A Bio-Rad protein assay reagent was used to determine the total protein concentration and equal amounts of cytosolic protein (20 µg) were assayed for their relative toxin level using *C. difficile* Tox A/B II ELISA kit from Tech Lab (VA, USA).

III. **Complementation of the R20291::*tcdR* mutant**

R20291 chromosomal DNA was used to PCR amplify the *tcdR* ORF along with its upstream DNA using primers ORG403 and ORG209 (Table 2), which carried restriction sites *KpnI* and *BamHI* respectively. The resulting PCR product and the vector pRPF185 were digested with *KpnI*, *BamHI* and ligated to yield pRGL294 (Table 1). The empty vector or the pRGL294 was introduced into *C. difficile* R20291::*tcdR* by conjugation. Transconjugants were grown overnight in TY medium supplemented with thiamphenicol (15 µg/ml). One hundred microliters of overnight culture was used to initiate fresh 10 ml cultures. The 10 h old culture was used to measure the toxins.

**IV. RNA seq analysis**

Total RNA was isolated from three biological replicates of each strain belonging to late stationary phase (18 hours after inoculation) and quality was checked using Agilent 2100 Bioanalyzer. The rRNA content in the selected samples was depleted using Epicenter Bacterial Ribo-Zero kit. The depleted rRNA fraction was used to construct strand specific single end cDNA libraries according to manufacturers’ instructions (using Truseq Small Stranded Total RNA sample prep kit, Illumina). Illumina HiSeq2000 sequencer (multiplexing 3 samples per lane) was used to sequence libraries. Sequences were cleaned (AlienTrimmer [61]) of adapter sequences, low quality sequences and only sequences with a minimum of 30 nucleotide in length was considered for further analysis. Out of three biological replicates, based on the quality of the data, only two were qualified for statistical analysis. Cleaned genes were aligned to reference genome (FN545816.1) using Bowtie (version 1.0.1) [60]. DESeq2 version 1.8.3 was used to perform normalization and differential analysis. Genes were considered differentially expressed if the fold change was ≥ 2.0 and their adjusted *p* value is ≤0.05. The mapped reads were formatted and visualized using COV2HTML [62] (https://mmonot.eu/COV2HTML/visualisation.php?str_id=-40). RNA-Seq data have been deposited in NCBI-GEO with accession n° GSE85395.

**V. Quantitative reverse transcription PCR (qRT-PCR)**

*C. difficile* R20291 and R20291*::tcdR* cultures were grown in TY or in 70:30 medium and cells were harvested at 16 h and 24 h by centrifugation at 4 °C for 2 min. Total RNA was extracted from the harvested cells following the protocol described previously [[9](#_ENREF_9), 60] and treated with DNase (Turbo; Ambion) for 30 min at 37 °C. 30 µL of final reaction volume comprising of 5 µg of template RNA, 4 µL of deoxynucleoside triphosphates (dNTP; 10mM each), 1 µg of hexamer oligonucleotide primer (5 µg/µL pdN_6_; Roche), and 6 µL of reverse transcription (RT) buffer was heated at 80 °C for 5 min and cDNA was synthesized at 42 °C for 2 hours using avian myeloblastosis virus (AMV) reverse transcriptase (Promega). Final 20 µL reaction volume containing 10 ng or 10 pg (for 16S rRNA) of cDNA, 400 nM gene-specific primers, and 12.75 µL of SYBR PCR master mix (BioRad) was used to perform Real-time quantitative PCR using iQPCR real-time PCR instrument (BioRad). Amplification and detection was performed as described previously [[9](#_ENREF_9)]. Quantity of cDNA of a gene in each sample was normalized to the quantity of *C. difficile* 16S rRNA gene and the ratio of normalized target concentrations (threshold cycle [2^−ΔΔCt^] method) [[9](#_ENREF_9), 55] gives the relative change in gene expression.
